# HIV Seroprevalence among Tuberculosis Patients in India, 2006–2007

**DOI:** 10.1371/journal.pone.0002970

**Published:** 2008-08-20

**Authors:** Neeraj Raizada, Lakbir Singh Chauhan, Ajay Khera, Jotna Sokhey, D. Fraser Wares, Suvanand Sahu, Rahul Thakur, Puneet Kumar Dewan

**Affiliations:** 1 Office of the WHO Representative to India, New Delhi, India; 2 Central Tuberculosis Division, Directorate General of Health Services, New Delhi, India; 3 Ministry of Health and Family Welfare, National AIDS Control Organization, Ministry of Health and Family Welfare, New Delhi, India; 4 World Health Organization, Southeast Asia Regional Office, New Delhi, India; National AIDS Research Institute, India

## Abstract

**Background:**

Little information exists regarding the burden of HIV among tuberculosis patients in India, and no population-based surveys have been previously reported. A community-based HIV prevalence survey was conducted among tuberculosis patients treated by the national tuberculosis control programme to evaluate the HIV prevalence among tuberculosis patients in India.

**Methodology/Principal Findings:**

Fifteen districts (total population: 40.2 million) across 8 states were stratified by HIV prevalence in antenatal clinic HIV surveillance sites and randomly selected. From December 2006 to May 2007, remnant serum was collected from patients' clinical specimens taken after 2 months of anti-tuberculosis treatment and subjected to anonymous, unlinked HIV testing. Specimens were obtained and successfully tested for 5,995 (73%) of 8,217 tuberculosis patients eligible for the survey. HIV prevalence ranged widely among the 15 surveyed districts, from 1% in Koch Bihar, West Bengal, to 13.8% in Guntur, Andhra Pradesh. HIV infection was 1.3 times more likely among male TB patients than among female patients. Relative to smear-positive tuberculosis, HIV infection was 1.4 times more likely among smear-negative patients and 1.3 times more likely among extrapulmonary patients. In 4 higher-HIV prevalence districts, which had been previously surveyed in 2005–2006, no significant change in HIV prevalence was detected.

**Conclusions:**

The burden of HIV among tuberculosis patients varies widely in India. Programme efforts to implement comprehensive TB-HIV services should be targeted to areas with the highest HIV burden. Surveillance through routine reporting or special surveys is necessary to detect areas requiring intensification of TB-HIV collaborative activities.

## Introduction

The harmful synergy between the HIV and tuberculosis epidemics has added dramatically to the suffering and death caused by each disease alone[1]. HIV-infection is among the strongest risk factors for progression of latent tuberculosis infection to active disease[2], [3]. HIV surveillance among tuberculosis patients allows assessment of the impact of the HIV epidemic on the tuberculosis situation and facilitates planning of collaborative activities between HIV/AIDS and tuberculosis programmes. Furthermore, surveillance provides information necessary to monitor the effectiveness of joint strategies aimed at reducing the impact of HIV among tuberculosis patients[4].

India has the highest total burden of tuberculosis in the world—with an estimated 1.85 million incident cases in 2005[5]. The effect, however, of the HIV epidemic on TB in India is not understood by most. The National AIDS Control Organization (NACO) estimates that 2.47 million persons (approximately 0.36% of the adult population) were living with HIV infection in India in 2006. The distribution of HIV, however, is highly heterogeneous, and HIV prevalence may be increasing in some areas, while stable or decreasing in others[6]. For several years, anecdotal reports from referral institutions in India have suggested that HIV prevalence is high among TB patients[7]–[14]. These findings cannot be generalized to tuberculosis patients diagnosed and treated through community-based services.

Tuberculosis control services in India are provided through the Revised National Tuberculosis Control Programme (RNTCP), which in 2005 reported and treated 1.16 million incident tuberculosis cases[15]. The first round of population-based surveillance of HIV infection in tuberculosis patients using RNTCP services was conducted in 2005–6 in four districts with high HIV-prevalence in South India[16]. To guide collaborative TB-HIV activities, the government of India expanded the HIV surveillance of tuberculosis patients to 15 districts during 2006–2007, covering districts in different stages of the HIV epidemic.

## Methods

### District selection

**Figure 1:**
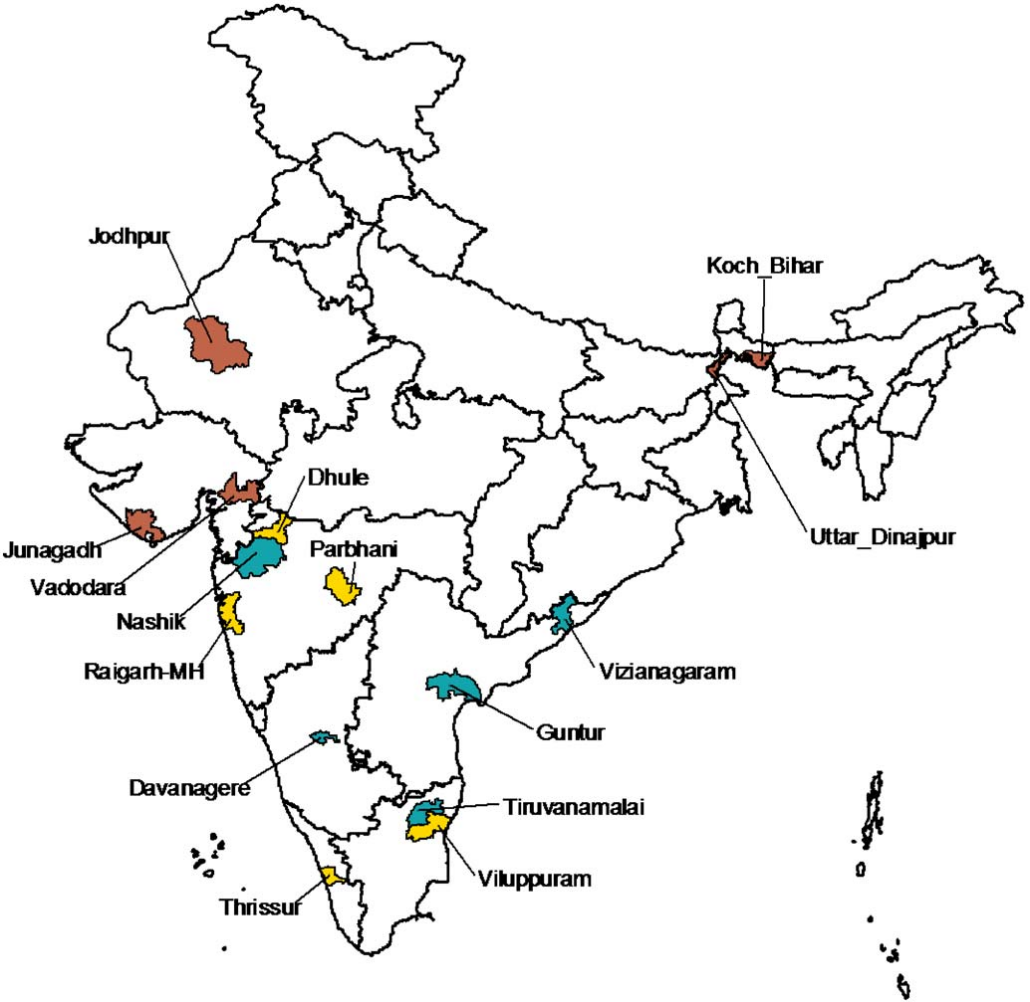
Districts selected for the survey. District selection stratified by mean HIV seroprevalence in antenatal clinic (ANC) surveillance sites, 2003–2005. Districts shaded blue had mean 2003–2005 ANC HIV seroprevalence 0–0.5%, yellow districts had ANC HIV seroprevalence 0.51–1.0%, and red districts had ANC HIV seroprevalence >1.0%. The districts of Davangere, Guntur, Nasik, and Tiruvanamalai were selected in the previous years' survey, and were purposively selected again for trend analysis.

Operational and supervision constraints limited the total number of districts surveyed to 15. Sentinel districts were selected with the *a priori* understanding that district results could not be generalized to the state or national level. District selection was stratified by antenatal clinic (ANC) HIV seroprevalence using sentinel surveillance data to allow future trend evaluation in settings with different HIV/AIDS epidemiological situations[16]. We selected 5 of 72 districts with a mean 2003–2005 antenatal HIV seroprevalence of 0–0.5%, and 5 of 59 districts with a mean 2003–2005 antenatal HIV seroprevalence of 0.51–1.0% (Fig. 1). Among the 79 districts with an ANC HIV seroprevalence >1%, 1 district was randomly selected in addition to 4 districts previously chosen randomly for the 2005–2006 survey; these 4 were included again for the purpose of trend analysis. The total 2006 population of the districts selected was 40.2 million persons.

### Patient eligibility and enrollment

All notified new cases of tuberculosis, (pulmonary and extrapulmonary) in persons ≥14 years old were eligible. Eligibility was limited to patients newly diagnosed with tuberculosis to preclude the possibility of double-counting cases. Patients were selected consecutively from RNTCP-designated microscopy centers (DMCs) located in public clinics and hospitals in each district. Patients were enrolled at their routine 2-month follow up visit to the DMC as this enabled the inclusion of smear-negative and extrapulmonary TB cases. Enrollment at the follow-up visit also ensured that only confirmed tuberculosis cases were included. Of the 460 DMCs in the survey districts, 150 DMCs with <10 tuberculosis cases per quarter were excluded from the survey for operational considerations.

A sample size of 400 tuberculosis patients per district was selected, based on the minimum number of patients needed to detect a prevalence of at least 5% HIV infection, with 95% confidence and 40% precision.

### HIV testing

At the time of their 2-month follow-up visit, all eligible tuberculosis patients were offered a free, voluntary liver function test (LFT). If patients verbally consented to specimen collection for LFT, samples were collected at the DMC; refusals were documented. LFT test results were communicated back to the provider. Remnant serum specimens were used for unlinked, anonymous HIV testing.

To ensure uniform implementation across all 15 districts, standard training material and operating procedures were developed, and trainings conducted at all sites by a single training team. Supervision checklists were developed for local programme supervisors, and progress was tracked by bi-weekly reports.

### Ethical issues

This surveillance activity was conducted after review and approval of NACO and the Central Tuberculosis Division, Directorate General of Health Services, Ministry of Health and Family Welfare, Government of India. The surveillance activity was justified as necessary to develop and guide national TB and HIV programme policies. It was felt that the information could not be accurately obtained by surveillance methods other than anonymous, unlinked testing. Due to the anonymous, unlinked design, the activity relied on testing of remnant specimens collected for another clinical purpose. HIV results were not individually identifiable, and hence could not be returned. Patients were not asked to provide specific consent for the anonymous, unlinked HIV testing of their specimen remnants. However, free voluntary HIV counseling and testing was provided for all TB patients as per national guidelines[17], and all HIV-infected patients were eligible for free care, including anti-retroviral treatment, through the National AIDS Control Programme. This approach to HIV surveillance among TB patients has been included in WHO guidelines[4] and is in line with current National AIDS programme HIV surveillance strategy.

### Laboratory methods

The DMC laboratory technicians received special training on blood sample collection, serum separation, storage and the transportation of samples to testing sites. HIV testing was conducted by a quality-assured laboratory network established by NACO for HIV surveillance. An unidentified refrigerated aliquot of serum was transported from the collection site to the designated laboratory within 7 days of collection. Initial HIV testing was performed by the Enzyme-Linked ImmunoSorbent Assay (ELISA). Specimens reactive on the first assay were retested with a rapid assay, and results were interpreted as HIV-positive only if both tests were reactive. All the HIV-reactive serum and 5% of the HIV-negative serum samples were sent to national reference laboratories for re-testing per routine NACO quality assurance procedures.

### Data collection & analysis

Data were double entered and validated against the original test reports, and analyzed with EpiData v1.1 (EpiData Association, Odense, Denmark). We calculated the HIV prevalence among all TB patients in each district. We also calculated a standardized HIV prevalence rate to account for the under-enrollment of smear-negative and extrapulmonary tuberculosis cases in several districts. To standardize the HIV prevalence rate, the tuberculosis-type specific HIV prevalence was applied to the number of eligible tuberculosis cases for each type of tuberculosis for each district. This tuberculosis-type standardized HIV prevalence was compared to the non standardized prevalence. Proportions were compared using a chi-square test, or Fisher's exact test if any number was <5. Confidence intervals were calculated by the Wilson score method[18].

## Results

### Enrollment

**Figure 2:**
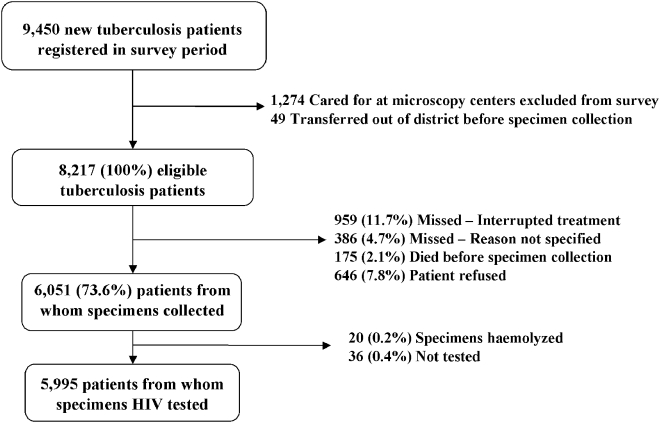
Survey enrollment and inclusion, with reasons for non-eligibility and non-enrollment into survey.

Of the 9,450 new tuberculosis patients registered during the survey period, 1,274 were seen at excluded microscopy centers and classified as ineligible (Fig. 2). Excluding 49 patients who transferred out of the survey area before specimen collection, 8,217 tuberculosis patients in the 15 districts fulfilled the eligibility criteria. Of these 2,166 were not included—most commonly because of treatment interruptions (959 persons, 11.7%) or refusal (646 persons, 7.8%). Serum specimens were collected from 6,051 (73.6%) of the eligible tuberculosis patients, and HIV test results were available for 5,995 (73.0%) of the eligible patients.

### Patient characteristics

Among the 5,995 patients tested in the survey, the median age was 35 years (range 14–95 years), and 3998 (69.4%) were male patients. No significant difference existed between districts in the age or sex distribution of tested patients. Overall, 3395 (56.6%) of tested patients were smear-positive, 1725 (28.8%) were smear-negative, and 866 (14.4%) had only extra-pulmonary tuberculosis; type of tuberculosis was not reported for 9 (0.2%) patients.

Compared to patients not included in the survey, no significant difference existed in the distribution of age or sex. However, in 7 districts, there was a higher proportion of smear-negative and/or extrapulmonary tuberculosis among the patients who were not included.

### HIV prevalence results

HIV infection was detected in tuberculosis patients in all 15 surveyed districts. HIV seroprevalence among tuberculosis patients ranged widely, from 1% in Koch Bihar to 13.8% in Guntur (Table 1). In the aggregate population of tested patients across all districts, HIV seroprevalence in tuberculosis patients was highest in the groups of those aged 25–34 years (11.0%) and 35–44 years (10.6%). However, instances of HIV infection were observed in tuberculosis patients up to 70 years. HIV seroprevalence was higher among male tuberculosis patients than female tuberculosis patients (8.4% vs. 5.6%, relative risk [RR] 1.28, 95% confidence interval [CI] 1.04–1.59). Compared to smear-positive tuberculosis cases (5.8% HIV seroprevalence), the survey detected a higher HIV prevalence among smear-negative (8%; RR: 1.41, 95% CI, 1.13–1.76) and extrapulmonary tuberculosis cases (7.4%; RR: 1.30, 95% CI, 0.96–1.72). In four districts where the survey was also conducted during 2005–2006, no significant difference in HIV prevalence in tuberculosis patients was observed over 2 years (Table 2).

**Table 1:** HIV seroprevalence among tuberculosis patients in 15 districts in India, 2006–2007.

State	District	HIV stratification^a^	Tested	HIV-positive	HIV seroprevalence	95% Confidence Interval
Andhra Pradesh	Guntur	High	400	55	13.8%	10.7–17.5%
	Vizianagaram	Medium	399	26	6.5%	4.5–9.4%
Gujarat	Junagadh	Low	399	16	4.0%	2.5–6.4%
	Vadodara	Low	399	10	2.5%	1.4–4.6%
Karnataka	Davanagere	High	400	37	9.3%	6.8–12.5%
Kerala	Thrissur	Medium	402	22	5.5%	3.6–8.2%
Maharashtra	Dhule	Medium	400	44	11.0%	8.3–14.5
	Nashik	High	400	16	4.0%	2.5–6.4%
	Parbhani	Medium	400	48	12.0%	9.2–15.6%
	Raigarh	Medium	401	33	8.2%	5.9–11.3%
Rajasthan	Jodhpur	Low	400	11	2.8%	1.5–4.9%
Tamil Nadu	Tiruvanamalai	High	399	37	9.3%	6.8–12.5%
	Villipuram	Medium	401	31	7.7%	5.5–10.8%
West Bengal	Koch Bihar	Low	394	4	1.0%	0.4–2.6%
	Uttar Dinajpur	Low	401	9	2.2%	1.2–4.2%

a Districts stratified by mean HIV seroprevalence in antenatal clinic (ANC) surveillance sites, 2003–2005. Low <0.5%, Medium = 0.51–1.0%, High >1.0%.

**Table 2:** Trends in HIV seroprevalence among tuberculosis patients in 4 districts, 2005–6 survey and 2006–7 surveys.

District	Survey year	Number Tested	Number HIV-positive	Percent	95% CI
Davangere	2005–2006	400	38	9.5%	7.0–12.8%
	2006–2007	400	37	9.3%	6.8–12.5%
Guntur	2005–2006	400	64	16.0%	12.7–19.9%
	2006–2007	400	55	13.8%	10.7–17.5%
Nashik	2005–2006	400	17	4.3%	2.7–6.7%
	2006–2007	400	16	4.0%	2.5–6.4%
Thiruvanamalai	2005–2006	400	25	6.3%	4.3–9.1%
	2006–2007	400	37	9.3%	6.8–12.5%

To evaluate whether differences in the distribution of TB type in patients tested and those not included in the survey led to an underestimation of HIV prevalence in tuberculosis patients, we compared the calculated prevalence with the tuberculosis-type standardized HIV prevalence in each district. There were no significant differences between standardized and nonstandardized HIV prevalence in any district.

## Discussion

This survey represents the first reported HIV-prevalence data from a community-based survey among tuberculosis patients in India. The survey identified a wide distribution of HIV seroprevalence among tuberculosis patients, ranging from 1% to 13.8% in the 15 surveyed districts. These data suggest that HIV-infection among tuberculosis patients may exist everywhere, but the wide distribution in severity will create operational challenges for the design and implementation of collaborative TB-HIV interventions.

This survey has important implications for the Indian tuberculosis and HIV control programmes. The prevalence of HIV among tuberculosis patients exceeded 5% in 8 of 9 districts from states considered to have high HIV prevalence (Andhra Pradesh, Karnataka, Maharashtra, and Tamil Nadu). Five percent is the threshold at which WHO has recommended intensified interventions to address TB-HIV, including voluntary HIV testing of all tuberculosis patients[19]. This finding supports the recent decision of the Ministry of Health and Family Welfare to adopt the policy of routinely offering voluntary HIV counseling and testing to all tuberculosis patients in states with higher HIV burdens[20].

Conversely, in low-prevalence areas where more than 95% of TB patients are HIV-negative, a uniform policy of testing all tuberculosis patients for HIV may generate substantial operational difficulties while yielding little improvement in health outcomes. Quality-assured HIV counseling and testing services are usually only available in urban sections in low-prevalence areas[21]. Until HIV testing services are made available at the sub-district level to match the widespread availability of tuberculosis services, in these settings the preferable course may be selective referral, based on risk factors or clinical signs of HIV infection.

In the aggregate population of all tested patients, a few general trends of HIV epidemiology among tuberculosis patients emerged. HIV-infection was more common in tuberculosis patients aged 25–44 years, but was found in all age groups in the surveyed population and in patients up to age 70 years. In tuberculosis patients, HIV-infection was more common among males patients than female patients, as has been observed in the general population[6]. Also, compared to new smear-positive pulmonary TB patients, HIV-infection was 1.4 and 1.3 times more common among smear-negative and extrapulmonary patients respectively. These observations are consistent with what is known about HIV epidemiology in general in India and the clinical presentation of tuberculosis in HIV-infected persons[6], [22].

In the 4 districts with repeat surveys, no significant change was detected in HIV prevalence among tuberculosis patients. This finding is reassuring and adds to the data suggesting stability in the HIV epidemic in India. However, these 4 districts were considered to have high HIV prevalence as judged by the mean 2003–2005 ANC HIV seroprevalence >1% at sentinel surveillance sites. No trend data is available for areas with lower HIV prevalence.

What is the optimal future method of conducting HIV surveillance among TB patients in India? Given the epidemiological diversity demonstrated in this survey and the geographical vastness of India, special surveys may not be able to generate the necessary local information needed to guide tuberculosis and HIV programmes. Instead, routine recording and reporting of HIV status of tuberculosis patients by the tuberculosis programme is probably the preferable surveillance option in high HIV prevalence states. Periodic HIV surveillance among tuberculosis patients may still have some value in low HIV prevalence states to detect when intensification of TB-HIV collaborative activities is indicated, or as a sentinel activity to detect early increases in community HIV seroprevalence.

### Limitations

This survey was not intended to be generalized to the state or national levels. Because of a number of limitations, this survey should be considered cautiously as a minimum estimate, and the actual prevalence of HIV infection among all tuberculosis patients in these districts may be slightly higher. First, only new tuberculosis patients were enrolled to limit the possibility of double-counting persons who failed treatment and were re-registered as re-treatment cases. Recurrent tuberculosis is known to be more common among HIV-infected persons, and this may lead to some underestimation of HIV prevalence among all tuberculosis patients[23]. However, in the 15 districts surveyed, only 12% of patients in the 1st and 2nd quarter of 2007 were registered as re-treatment cases; unless the difference in HIV prevalence between new and re-treatment tuberculosis cases were very large, this is not likely to have substantially influenced our results.

Specimens were collected from patients during the 2^nd^-month clinical evaluation. There were 175 patients, however, who died and 959 who interrupted treatment during the first 2 months and could not be included. Death during tuberculosis treatment is known to be higher among HIV-infected persons than non–HIV-infected persons. It is unknown if tuberculosis treatment interruptions are more common among HIV-infected persons than non–HIV- infected persons in India, but some deaths may have been classified as treatment interruptions. If the prevalence of HIV infection were greater in this unincluded group, then we may have underestimated the HIV prevalence among tuberculosis patients in this survey.

Only cases that were notified to the RNTCP were included in the survey. Limited data about patients treated outside the government programme is available to estimate the burden of tuberculosis or the HIV prevalence among those with tuberculosis. No data suggests that the HIV prevalence would be substantially different than those notified to the tuberculosis programme, particularly with the large-scale involvement of public and private medical colleges in the tuberculosis programme over the past 2 years[24].

### Conclusions

The survey demonstrated a diverse distribution of HIV infection among tuberculosis patients in India, which supports targeted programme efforts. Periodic surveys may have a role in areas with low HIV prevalence as a tool to help detect emerging pockets of HIV infection and guide future HIV prevention efforts. Future surveillance of HIV infection in tuberculosis patients in India may be based on routine data collected by the tuberculosis programme recording and recording system, coupled with programme efforts to improve the uptake of HIV counseling and testing in this patient population. The association between HIV prevalence among tuberculosis patients, HIV surveillance at antenatal clinic, and community-based surveys requires further investigation.
